# Assessing plastic ingestion in birds of prey from British Columbia, Canada

**DOI:** 10.1007/s11356-023-27830-4

**Published:** 2023-05-27

**Authors:** Kerry Schutten, Akshaya Chandrashekar, Madelaine Bourdages, Victoria Bowes, John Elliott, Sandi Lee, Tony Redford, Jennifer Provencher, Claire Jardine, Laurie Wilson

**Affiliations:** 1 Department of Pathobiology, Ontario Veterinary College, University of Guelph, 50 Stone Rd E, Guelph, ON N1G 2W1 Canada; 2 Carleton University, Ottawa, ON Canada; 3 Government of British Columbia, Abbotsford, British Columbia Canada; 4 Environment and Climate Change Canada, Ottawa, Canada

**Keywords:** Raptors, Anthropogenic, Debris, Pollution, Susceptibility, Vulnerability, Scavengers

## Abstract

Since first being introduced for public use in the 1960s, plastic has become one of the most pervasive and ubiquitous forms of pollution globally. The potential fate and effects of plastic pollution on birds is a rapidly growing area of research, but knowledge of terrestrial and freshwater species is limited. Birds of prey have been particularly understudied, with no published data on plastic ingestion in raptors in Canada to date, and very few studies globally. To assess the ingestion of plastic in raptors, we analysed the contents of the upper gastrointestinal tracts from a total of 234 individuals across 15 raptor species, collected between 2013 and 2021. Upper gastrointestinal tracts were assessed for plastics and anthropogenic particles > 2 mm in size. Of the 234 specimens examined, only five individuals across two species had evidence of retained anthropogenic particles in the upper gastrointestinal tract. Two of 33 bald eagles (*Haliaeetus leucocephalus*, 6.1%) had retained plastics in the gizzard, while three of 108 barred owls (*Strix varia*, 2.8%) had retained plastic and non-plastic anthropogenic litter. The remaining 13 species were negative for particles > 2 mm in size (*N* = 1–25). These results suggest that most hunting raptor species do not appear to ingest and retain larger anthropogenic particles, though foraging guild and habitat may influence risk. We recommend that future research investigate microplastic accumulation in raptors, in order to gain a more holistic understanding of plastic ingestion in these species. Future work should also focus on increasing sample sizes across all species to improve the ability to assess landscape- and species-level factors that influence vulnerability and susceptibility of plastic pollution ingestion.

## Introduction

Since its initial widespread use began in the 1960s, plastic has become one of the most pervasive and ubiquitous forms of pollution globally, and has been identified alongside climate change as an emerging environmental problem with the potential to greatly impact human and environmental health (Blettler et al. 2017; UNEP 2014). In 2014, Eriksen et al. estimated that more than 5 trillion plastic particles were floating in the world’s oceans, while in 2015, van Sebille et al. (2015) estimated the marine microplastic particle burden weighs up to 236 thousand metric tonnes. Despite these already alarming numbers, most researchers consider any estimates of plastic waste to be extremely conservative (Puskic et al. 2020). Ingestion of plastics has been demonstrated in birds across hundreds of species globally, and in varying habitats, with a large increase in avian plastic ingestion studies reported in recent years (Provencher et al. 2017; Wang et al. 2021). Seabirds are the most commonly studied group of birds, in part due to characteristics that make them ideal for studying, such as high breeding site fidelity and large colony numbers which provide an ideal opportunity for repeated and robust sampling (Carlin et al. 2020; Poon et al. 2017; Provencher et al. 2019). However, there is a demonstrated need for research in non-marine environments and in understudied species (Carlin et al. 2020; Jagiello et al. 2019; Wang et al. 2021). A baseline understanding of plastic ingestion in terrestrial and freshwater environments is critical not only to understanding health risks and conservation priorities but also for identifying highly exposed species that can act as sentinels for environmental monitoring (Provencher et al. 2015).

Raptors, also known as birds of prey, are considered an important group for research given the potential range of exposure across foraging niches and trophic levels (Wang et al. 2021). McClure et al. (2019) defined raptors as “species within orders that evolved from raptorial land birds (telluraves), in which most species maintained raptorial lifestyles as derived from their common ancestor”, and argue that this definition is most inclusive of the overlapping phylogenetic, morphological, and ecological traits associated with hawks, eagles, kites, owls, falcons, and vultures (New and Old World). Globally, the few studies on plastic ingestion in terrestrial birds have focused mainly on obligate scavengers, such as vultures, as these species are considered to be at higher risk of exposure to plastic and other anthropogenic waste due to their synanthropic behaviour (e.g. Augé 2017; Ballejo et al. 2021; Cunha et al. 2022; Plaza and Lambertucci 2017). Ingestion of plastic in these species may be intentional or accidental, with low acceptance threshold and high cue overlap of plastics with acceptable prey items making these species more susceptible to plastic ingestion. High abundance of plastic is common in scavenging sites such as landfills, making these species more vulnerable to plastic ingestion (Santos et al. 2021; Wang et al. 2021).

There is little information to date on plastic ingestion in birds of prey that are primarily raptorial predators, despite evidence that some of these species are also opportunistic scavengers that will utilise anthropogenic sources of food (e.g. Bouker et al. 2021), or could be otherwise exposed to plastic ingestion through trophic transfer (Carlin et al. 2020). Carlin et al. (2020) quantified the abundance of plastics throughout the entire gastrointestinal tract in eight bird of prey species, which included hawks, owls, osprey, and vultures. Sample sizes ranged from 1 to 28, making comparisons across species difficult. Only the stomachs of black vultures (*Coragyps atratus*) contained macroplastics (> 5 mm), but all species contained microplastics, and red-shouldered hawks (*Buteo lineatus*) contained the greatest mean number of microplastics per gram in the gastrointestinal tract (Carlin et al. 2020). While birds of prey share many common characteristics, they still demonstrate a wide variety of foraging strategies, dietary preferences, and geographic ranges. A meta-analysis of plastic ingestion data for almost 50,000 seabirds demonstrated that foraging method and diet are significant predictors for plastic ingestion in these species, and similar drivers may influence plastic ingestion risk in birds of prey (Avery-Gomm 2020). Evidence is also mounting that scavenging species like raptors are at high risk of plastic exposure and ingestion due to this foraging strategy (i.e. Ballejo et al. 2021; Wang et al. 2021), and that diet preference may be a driving mechanism for differences in microplastic accumulation across raptor species (Carlin et al. 2020).

To investigate whether birds of prey are ingesting and retaining plastics and other anthropogenic particles, we examined the stomach contents of 15 species from British Columbia (B.C.), Canada. Our first objective was to assess ingestion of visible plastics and other anthropogenic particles (> 2 mm in size) in raptor species that were collected as part of passive wildlife health surveillance. We predicted that birds of prey would rarely ingest and retain visible litter (macroplastics and larger microplastics), based on dietary preferences that reduce the risk of plastic being mistaken for prey. The second objective was to assess whether litter ingestion varied across bird-level factors such as species and foraging niche. We predicted that ingestion of plastic would be more likely in species like bald eagles (*Haliaeetus leucocephalus*), which are opportunistic scavengers, compared to obligate hunters such as owl species. The final objective was to assess whether birds of prey could be used as sentinel species for monitoring litter in the environment. We did not expect that most raptor species would make ideal indicators due to low species susceptibility to ingestion. However, scavenger species that are more likely to ingest plastic could represent opportunities for environmental plastic monitoring.

## Materials and methods

### Sample collection

Between 2013 and 2021, a total of 234 individuals across 15 species were opportunistically collected from a variety of organisations as part of a broader wildlife disease surveillance strategy in British Columbia, Canada, and submitted for post-mortem examination by wildlife pathologists. All collections were completed with appropriate permits and approval (British Columbia Wildlife Act permits SU12-76,336 and SU16-24,223; Environment Canada scientific salvage permits BC-SA-0020–13 through 18, SC-BC-2019-0012SAL, SC-BC-2020-0012SAL, and SC-BC-2021-0012SAL).

A routine post-mortem investigation was performed on each individual, with morphometric and geographic information recorded, including the species, sex, age (when possible), and the location where each individual was initially located. The details of how each individual died were recorded (i.e. euthanasia, died in care, or dead on arrival to the facility), and the pathologist’s diagnoses of pathology or comorbidities were provided. Where possible, a definitive cause of death was noted by the pathologist. The distal oesophagus, proventriculus, and ventriculus (referred to collectively in this study as the stomach sample, or upper gastrointestinal tract, as per Provencher et al. 2018) were collected; in cases where further investigation to reach a diagnosis was warranted, the stomachs were opened and carefully examined for any obvious pathology before being frozen in individual bags with all stomach contents remaining in situ. Distal intestinal tracts were not saved as part of routine monitoring, and thus could not be utilised for plastic investigation in this study.

### Stomach content analysis

In 2020 and 2021, frozen stomach samples were shipped to Carleton University and the University of Guelph for plastics analysis. Once thawed, stomach contents were systematically analysed for anthropogenic particles > 2 mm (Provencher et al. 2017, 2019; van Franeker et al. 2011). Nested stainless steel sieves were used to capture particles > 2 mm in size and remove large pieces of organic material and liquids. Sediments were then removed using a 5 mmol/L NaCl solution as per Provencher et al. (2019). Litter particles were rinsed and dried, and then assessed systematically as per Provencher et al. (2017). Plastics were categorised as either industrial or user (fragment, foam, sheet, thread). Non-plastic particles of anthropogenic origin were labelled “other”, and length, weight (to nearest 0.001 g), and colour were described for all litter as per Provencher et al. (2017).

The size cut-off of > 2 mm was chosen based on necessity, for two reasons. Provencher et al. (2019) suggests 1 mm as a reasonable cut-off for visible microplastics in the stomachs of most bird species, as particles smaller than 1 mm are likely to pass through the sphincter, and thus not accumulate. We instead elected to use a 2-mm sieve due to convenience; arguably this cut-off is still likely to capture all particles large enough to accumulate, and the nature of digestible stomach contents for raptors (i.e. meat fibres) made it very challenging to separate organic material from small anthropogenic particles in a 1-mm gauge sieve. Secondly, due to the long-term nature of this project and the primary goal of determining cause of death for wildlife disease surveillance, stomachs were in some cases first opened for visual inspection by the pathologist and then frozen until the stomach content analysis occurred. Assessing for microplastics < 2 mm would have introduced more bias due to potential loss and/or contamination during the post mortem examination and, as such, sample analysis was restricted to visible debris. While this protocol has reduced the level of detail that can be provided from this analysis, its simplicity has instead allowed for a much broader geographic, species, and temporal scale due to ease of collection and long-term storage.

## Results

**Fig. 1:**
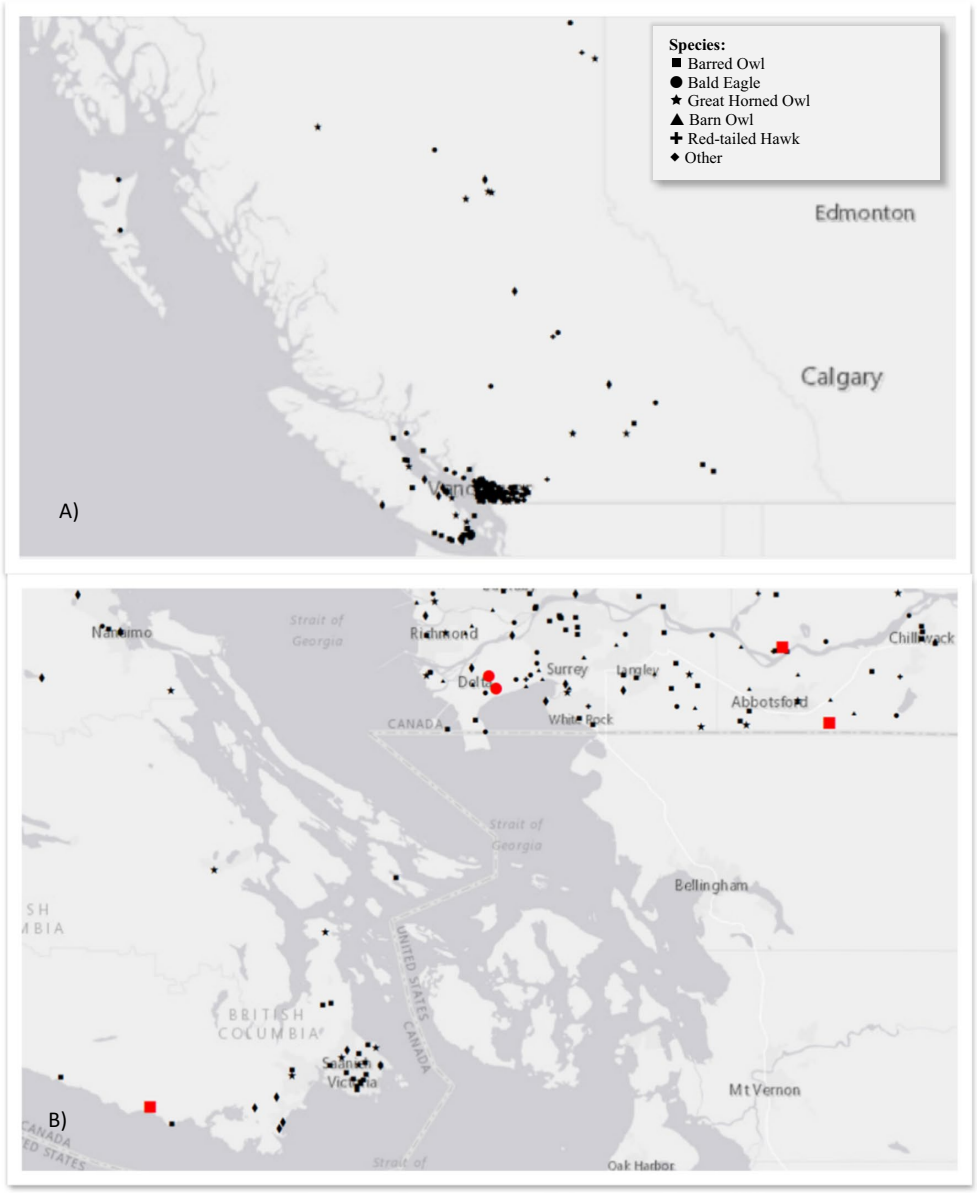
**A** Distribution of bird carcass locations by species across British Columbia (B.C.), Canada. Species where sample size was < 15 are included in “other.” **B** Inset, showing five bird carcasses which contained ingested anthropogenic particles highlighted in red, enlarged symbols. Note the two bald eagles are located in Delta, B.C., which is home to the Vancouver Landfill and Recycling Centre

**Fig. 2:**
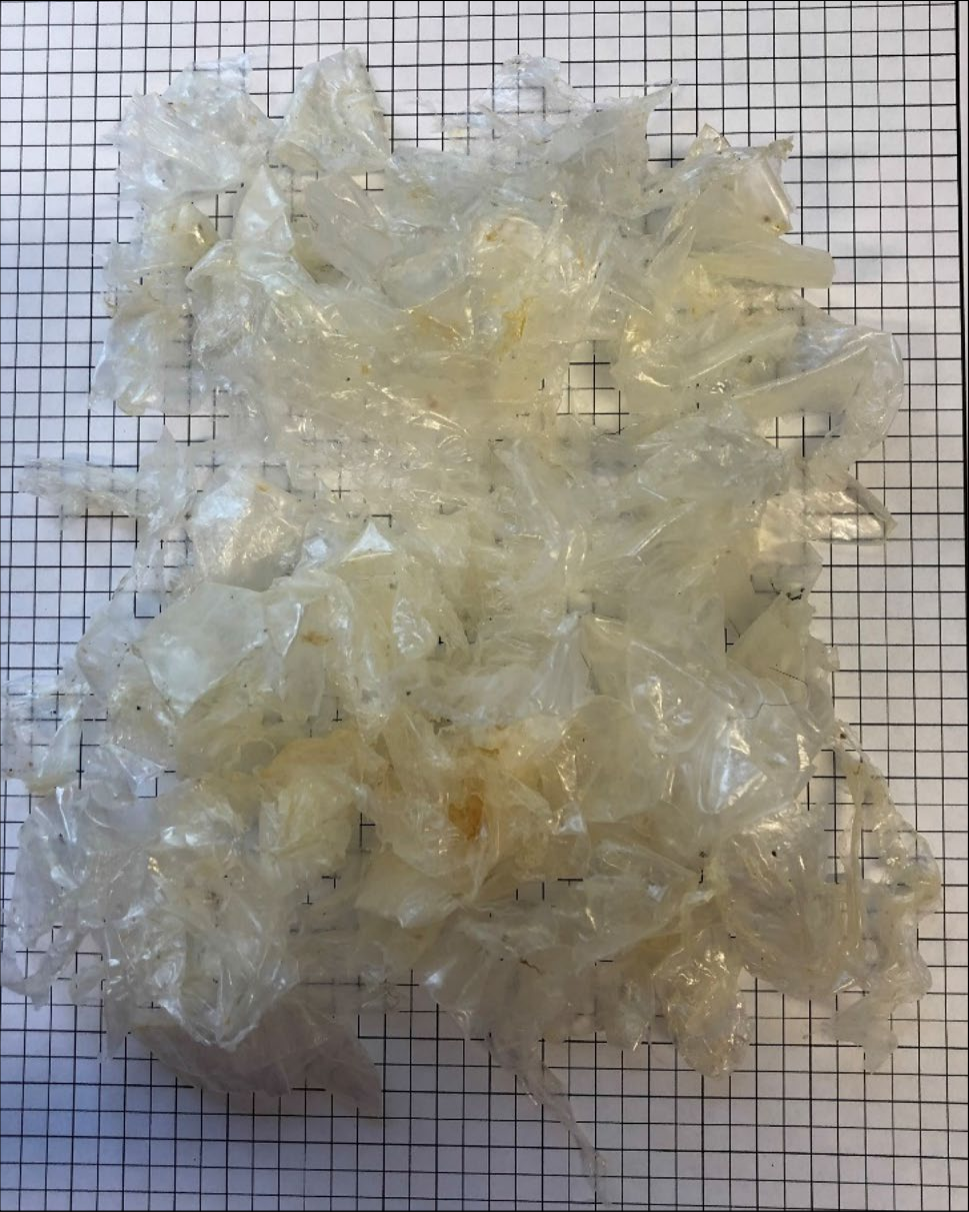
The accumulated sheet plastic found in the upper gastrointestinal tract of a subadult male bald eagle from Delta, British Columbia, Canada. There were 117 pieces of sheet plastic, 1 small thread, and 1 small foam pellet present, weighing a cumulative 11.1 g. Each square represents 5 mm

The species examined in this analysis included hawks, owls, falcons, and one turkey vulture (*Cathartes aura*) (Table 1). Individuals came from sites across British Columbia; however, the majority of birds were located in the metro-Vancouver area and Vancouver Island (Fig. 1A and B). Retained anthropogenic materials were found in only five of 234 individuals (% frequency of occurrence (FO) = 2.1) from two species: bald eagles (*N* = 2, % FO = 6.1) and barred owls (*Strix varia*) (*N* = 3, % FO = 2.8) (Table 2). The three barred owls each contained only a single small fragment of litter, while both bald eagles contained multiple pieces of plastic (Table 2). The first bald eagle had 119 individual pieces of clear to off-white sheet plastic weighing 11.1 g in total, which resembled the thickness and texture of a clear plastic garbage bag (Table 2, Fig. 2). The second bald eagle contained three similar pieces of plastic and one fragment of microfibre cloth, which resembled a cleaning wipe or baby wipe. Both bald eagles were sub-adult males from Delta, B.C., and died secondary to trauma sustained from collisions (one with a car, one presumed with a plane). The adult male and adult female barred owl died secondary to trauma from vehicular and building collisions. The cause of death of the third barred owl, a juvenile male, could not be determined due to generalised autolysis, though haemorrhage in the distal intestine was noted. In all five cases, retained anthropogenic litter in the stomach appears to be an incidental finding; all individuals were in fair to good body condition, and there were no gross gastric lesions associated with the ingested particles.

**Table 1:** Demographics and anthropogenic particle ingestion by species of individual birds included in analysis. All individuals were collected in British Columbia, Canada, between 2013 and 2021

Species	# of individuals	Sex			# of individuals with ingested anthropogenic particles	% Frequency of occurrence of ingested particles by species
		*Female*	*Male*	*Unknown*		
Barred owl (*Strix varia*)	108	53	52	3	3	2.8
Bald eagle (*Haliaeetus leucocephalus*)	33	13	20	0	2	6.1
Great horned owl (*Bubo virginianus*)	25	15	9	1	0	0.0
Barn owl (*Tyto alba*)	24	8	14	2	0	0.0
Red-tailed hawk (*Buteo jamaicensis*)	16	8	4	4	0	0.0
Cooper’s hawk (*Accipiter cooperii*)	12	8	4	0	0	0.0
Northern saw-whet owl (*Aegolius acadicus*)	5	4	1	0	0	0.0
Sharp-shinned hawk (*Accipiter striatus*)	4	1	3	0	0	0.0
American kestrel *(Falco sparverius*)	1	0	1	0	0	0.0
Merlin (*Falco columbarius*)	1	1	0	0	0	0.0
Northern goshawk (*Accipiter gentilis*)	1	0	1	0	0	0.0
Osprey (*Pandion haliaetus*)	1	0	1	0	0	0.0
Long-eared owl (*Asio otus*)	1	1	0	0	0	0.0
Peregrine falcon (*Falco peregrinus*)	1	0	1	0	0	0.0
Turkey vulture (*Cathartes aura*)	1	0	1	0	0	0.0
***Totals***	234	112	112	10	5	2.1

**Table 2:** Descriptions of plastic and non-plastic anthropogenic particles retained in stomach samples of five of 234 birds of prey

Species	Total # of particles	Anthropogenic particles by type					Total mass (g)	Average length in mm (range)	Colours	“Other” description
		*Sheet*	*Thread*	*Foam*	*Fragment*	*Other*				
Barred owl	1	0	0	0	0	1	0.01	18 (N/A)	Grey-silver	Tin foil
Barred owl	1	0	0	0	0	1	0.01	10 (N/A)	Red-pink	Thread made of cloth/fibre
Barred owl	1	0	0	0	1	0	0.01	10 (N/A)	Orange-brown	
Bald eagle	119	117	1	1	0	0	11.06	37.8 (3–136)	Sheet, foam: off/white-clear	
									Thread: blue-purple	
Bald eagle	4	3	0	0	0	1	0.37	65 (20–120)	Sheet, other: off/white-clear	Microfibre cloth/tissue

## Discussion and conclusion

### Do raptors ingest and retain macro- and microparticles of anthropogenic litter > 2 mm in size?

Although the global understanding of plastic ingestion in raptors is limited, our results are consistent with the available literature which suggests that most species do not accumulate macroplastics (Carlin et al. 2020). We found that only two species of the 15 included had retained particles in the upper gastrointestinal tracts, and that percent frequency of occurrence of litter ingestion within both species was low. It is worth noting that 19 of the birds that died in care were hospitalised for more than 4 days before dying or being euthanised; it is therefore possible that plastics were excreted from the upper gastrointestinal tract while these individuals were in care, and the results discussed here may reflect an underrepresentation. In general, studies of retained debris in the upper gastrointestinal tract in birds reflect a snapshot in time of ingested plastics. Retention and transit times for gastrointestinal plastics are related to both species-specific traits as well as properties specific to the ingested plastics, and warrants further research (Provencher et al. 2017). Due to the opportunistic nature of this study design, we had few (< 20) samples from several species, making inferences about those species challenging. Notably, only a single turkey vulture was represented and was negative for plastic ingestion. Ballejo et al. (2021) reported that 24.5% of regurgitated boluses from turkey vultures contained macroplastics, and Augé (2017) reported up to 62% of boluses containing anthropogenic debris from this species. Determining an appropriate sample size necessary to detect plastics for species with unknown prevalence of plastic ingestion is challenging, as a much larger sample size will be required to capture positive samples where plastic ingestion prevalence is low (Provencher et al. 2015). Van Franeker and Meijboom (2002) used a power analysis to determine the ideal sample size required to monitor plastic pollution ingestion in northern fulmar (*Fulmarus glacialis*) and found that between 20 and 40 samples resulted in stable variances, with little additional information gained with > 40 samples. This cannot be applied directly to the raptors in this study due to species-specific differences in plastic ingestion risk, but supports cautious interpretation of results for species with < 20 individuals. However, for barred owls, bald eagles, great-horned owls (*Bubo virginianus*), and barn owls (*Tyto alba*), the larger sample sizes lend more confidence that our results reflect plastic ingestion more accurately (Table 1).

### How do species-level factors influence risk of plastic ingestion in raptors, and how does that influence their potential as sentinel species for plastic pollution monitoring?

The two species where litter ingestion was noted differ widely in foraging strategy. Both are generalist predators, but while barred owls hunt mainly ground-dwelling prey using hawking techniques (flying after their prey to capture it), bald eagles employ multiple foraging techniques (De Graaf et al. 1985). They are notably good piscivorous hunters, using foot-plunging techniques to capture live prey, but they are also opportunistic scavengers (De Graaf et al. 1985; Elliott et al. 2006; Peterson et al. 2001). Although the low prevalence of litter ingestion reported here prevents robust statistical analysis of risk factors, we hypothesise that species-level characteristics such as foraging technique and dietary preference influence the risk of plastic ingestion in these populations. Plastic ingestion has been reported repeatedly in other facultative and obligate scavenging raptor species. Bouker et al. (2021) investigated several caracara species feeding at an open air landfill in Argentina and found that 87% of fresh faecal samples contained microplastics. Opportunistic sampling of fresh caracara carcasses also revealed plastic impaction in the crop to be the presumed cause of death for one individual (Bouker et al. 2021). Similar to findings discussed above in turkey vultures, investigations in black vultures, Andean condors (*Vultur gryphus*), and California condors (*Gymnogyps californianus*) have repeatedly demonstrated high prevalence of ingested plastics (Ballejo et al. 2021; Borges-Ramírez et al. 2021; Carlin et al. 2020; Cunha et al. 2022).

In the metro-Vancouver area, bald eagles are regularly noted to be roosting and scavenging at the Vancouver Landfill and Recycling Centre, in Delta B.C. (Elliott et al. 2006). In 2020, this landfill accepted 71% of the annual municipal solid waste generated by the Metro Vancouver area, or approximately 699,000 tonnes (City of Vancouver 2021). Hundreds of eagles at a time have been observed at this open air dump, leading it to become a haven for local birding enthusiasts (Pynn 2016). The two bald eagles with stomach samples containing plastics reported in this study both died as a result of trauma relating to collisions in Delta, B.C. (Fig. 1B). A 2006 study of foraging behaviour of bald eagles at the Delta landfill determined that, for the majority of eagles, the landfill is used primarily as a roosting site, but that for a small proportion of subadults, refuse scavenging can make up a large portion of their diet (Elliott et al. 2006). Our results are consistent with this finding, as both eagles were subadult and contained large volumes of sheet plastic similar to that used in garbage bags (Fig. 2).

We hypothesize that opportunistic scavenger species like bald eagles are more susceptible to plastic ingestion risk than other hunting raptors, and that their vulnerability varies based on landscape features such as access to open air landfills. The words susceptibility and vulnerability are often used interchangeably, but convey different meanings; to borrow from the human health literature, a person’s susceptibility to a certain condition reflects physical or personal qualities such as genetic predisposition, while a person’s vulnerability reflects external factors that influence risk (i.e. occupational hazards) (Bell et al. 2013). Applying these terms to the study of plastic pollution in wildlife can be very useful, as it allows us to consider the different ways that plastic ingestion risk varies across species and landscapes. A species’ susceptibility to plastic ingestion reflects inherent physiological predispositions to both ingest and retain plastic, such as foraging strategy or stomach morphology. A species’ vulnerability to plastic ingestion instead refers to external factors that influence risk of interacting with plastics. For example, populations that live within highly polluted landscapes will be more vulnerable to plastic ingestion, simply because there is more of it around; within that polluted landscape, specific foraging guilds will be more susceptible to ingesting that plastic due to the evolutionary and physiological traits that govern their foraging. Understanding the factors that impact susceptibility and vulnerability is important in many aspects of plastic pollution research; it helps us to determine vulnerable species and systems that warrant further study, to identify species that are most susceptible for broader conservation initiatives, and to identify species that could be useful as indicators for plastic pollution and its potential associated health impacts (Avery-Gomm et al. 2018).

For population-level studies, considering what is driving ingestion risk helps us to understand how habitat relates to vulnerability, and how plastic pollution and its impacts on wildlife could vary spatially. Among more commonly studied bird species, the influences of foraging strategy and habitat on plastic ingestion risk have been well studied. Roman et al. (2019) demonstrated that among seabirds, taxonomic grouping is the single most important ecological driver of plastic ingestion, but that foraging strategy, diet, and range in relation to pollution hotspots are also important predictors. Particularly, their findings support previous research that surface feeders and those with a crustacean-rich diet are at the highest risk. Despite both northern fulmars and black-legged kittiwakes (*Rissa tridactyla*) being surface feeders, and therefore having similar vulnerability to plastic pollution, there is documented variation in litter ingestion between species (Baak et al. 2020; Poon et al. 2017). This is likely related to species-specific diet preferences and anatomy which influence susceptibility; northern fulmars do not regurgitate indigestible remains, and therefore may retain plastic longer than species that do habitually regurgitate (Poon et al. 2017). Caldwell et al. (2020) evaluated plastic ingestion rates as a function of foraging niche in two closely related species and found that the generalist herring gull (*Larus argentatus*) had a significantly larger niche size and a significantly higher rate of plastic ingestion compared to the great black-backed gulls (*Larus marinus*). The paucity of research on plastic ingestion in raptors means we have little understanding so far of what the biggest drivers of plastic ingestion risk are in these species.

This study provides baseline evidence that for many raptor species, ingestion risk of macroplastics and larger microplastics is low. Bald eagles, as predicted based on foraging guild, appear to be more susceptible to ingestion. Barred owls ingested more anthropogenic waste than predicted, and we hypothesize that landscape-level effects are driving differences in vulnerability for this species. Previous research has demonstrated that barred owl diet is influenced by the degree of urbanisation within their home ranges, with rat species making up an increasing proportion of their diet as urban development increased (Hindmarch and Elliott 2015). Rat species in other countries have also been documented to ingest plastic (Thrift et al. 2022); future work should consider the relationship between landscape level factors such as urbanisation and vulnerability of plastic ingestion in raptors, and the potential for trophic transfer in these species. This research suggests that most raptor species would not make effective sentinel species for monitoring health impacts from plastic pollution, but that studying individuals across species ranges is valuable to increase our understanding of how vulnerability to plastic changes across the landscape. Bald eagles may be reasonable indicators of plastic pollution monitoring, especially in human-dominated landscapes where opportunistic scavenging increases exposure. Future research should focus on microplastic accumulation throughout the gastrointestinal tracts of raptors, as these particles may represent a source for chronic exposure and secondary health impacts in these high trophic-level species (Brookson et al. 2019; Carlin et al. 2020; Santos et al. 2021). Finally, while our research suggests that raptors do not generally make great sentinel species for plastic pollution, publishing null values such as those reported in this study is still critical to developing a global understanding of how plastic pollution varies across different landscapes and species distributions.

### Conclusion

Overall, this study demonstrated that birds of prey do not typically ingest and retain visible anthropogenic particles in their upper gastrointestinal tracts. Sample size limits this conclusion for some species, and thus it should be applied cautiously. Bald eagles had the largest percent frequency of occurrence of plastic ingestion of any species, potentially due to species-level traits such as opportunistic scavenging behaviour, which may increase their susceptibility to plastic pollution ingestion. The opportunistic nature of this data collection limited the ability to study microparticle accumulation < 2 mm. We recommend that future research investigate microparticle accumulation in both the upper and lower gastrointestinal tracts of raptors, as well as how these species may excrete plastic pollution into their environments (via guano, bolus production, or nest building activity), in order to gain a more holistic understanding of plastic exposure in these species. In general, the raptor species studied here do not appear to make ideal indicator species for plastic pollution, but bald eagles demonstrated higher prevalence of ingestion and warrant further study. Future work should focus on increasing sample sizes across all species to improve the ability to assess landscape-level and species-level factors that influence vulnerability and susceptibility of plastic pollution ingestion.

## Data Availability

The datasets generated during and/or analysed during the current study are available from the corresponding author on reasonable request.
